# Comparison of Outcomes After Transcatheter *Versus* Surgical Repeat Mitral Valve Replacement

**DOI:** 10.21470/1678-9741-2021-0341

**Published:** 2023

**Authors:** Amr A. Arafat, Ashraf I. Zahra, Abdulaziz Alhossan, Haneen Alghosoon, Mohammad Alotaiby, Monirah A. Albabtain, Adam I. Adam, Khaled D. Algarni

**Affiliations:** 1 Adult Cardiac Surgery Department, Prince Sultan Cardiac Center, Riyadh, KSA.; 2 Cardiothoracic Surgery Department, Tanta University, Tanta, Egypt.; 3 Cardiothoracic Surgery Department, Shbeen Elkom Teaching Hospital, Shbeen Elkom, Egypt.; 4 Cardiac Research Center, Prince Sultan Cardiac Center, Riyadh, KSA.; 5 Adult Cardiology Department, Prince Sultan Cardiac Center, Riyadh, KSA.; 6 Cardiology Clinical Pharmacy Department, Prince Sultan Cardiac Centre, Riyadh, KSA.

**Keywords:** Transcatheter Aortic Valve Replacement, Mitral Valve, Survival, Intensive Care Units, Length of Stay

## Abstract

**Introduction:**

Repeat transcatheter mitral valve replacement (rTMVR) has emerged as a new option for the management of high-risk patients unsuitable for repeat surgical mitral valve replacement (rSMVR). The aim of this study was to compare hospital outcomes, survival, and reoperations after rTMVR *versus* surgical mitral valve replacement.

**Methods:**

We compared patients who underwent rTMVR (n=22) from 2017 to 2019 (Group 1) to patients who underwent rSMVR (n=98) with or without tricuspid valve surgery from 2009 to 2019 (Group 2). We excluded patients who underwent a concomitant transcatheter aortic valve replacement or other concomitant surgery.

**Results:**

Patients in Group 1 were significantly older (72.5 [67-78] *vs.* 57 [52-64] years, *P*<0.001). There was no diference in EuroSCORE II between groups (6.56 [5.47-8.04] *vs.* 6.74 [4.28-11.84], *P*=0.86). Implanted valve size was 26 (26-29) mm in Group 1 and 25 (25-27) mm in Group 2 (*P*=0.106). There was no diference in operative mortality between groups (*P*=0.46). However, intensive care unit (ICU) and hospital stays were shorter in Group 1 (*P*=0.03 and <0.001, respectively). NYHA class improved significantly in both groups at one year (*P*<0.001 for both groups). There was no group effect on survival (*P*=0.84) or cardiac readmission (*P*=0.26). However, reoperations were more frequent in Group 1 (*P*=0.01).

**Conclusion:**

Transcatheter mitral valve-in-valve could shorten ICU and hospital stay compared to rSMVR with a comparable mortality rate. rTMVR is a safe procedure; however, it has a higher risk of reoperation. rTMVR can be an option in selected high-risk patients.

## INTRODUCTION

Elderly and frail patients are more frequently submitted to reoperative cardiac surgery due to the aging of the population and the advancement of surgical techniques. At least 4% of patients who had a mitral valve repair or replacement will require repeat mitral valve surgery^[1,2]^. Despite the excellent results achieved after mitral valve repair^[2]^, re-repair may not be feasible in the second operation, and mitral valve replacement (MVR) is required^[3]^. Recent research showed marked improvement in repeat MVR outcomes, and the results were comparable to the primary MVR^[4]^. Although there is a marked improvement in the surgical outcomes of repeat surgical mitral valve replacement (rSMVR), several patients are not considered for surgery due to high surgical risk.

Repeat transcatheter mitral valve replacement (rTMVR) has emerged as a new option for managing high-risk patients. Early results of rTMVR were encouraging; however, the generalization of the technique to a lower-risk patient requires extensive studies^[5]^. In a benchmark study, Ejiofor et al. reported a 5% mortality for rSMVR after a previous mitral valve repair and 9% after a previous replacement. Long-term survival was lower in patients with prior replacement^[6]^.

Studies comparing clinical and echocardiographic outcomes after rSMVR and rTMVR are limited, and no randomized trials were performed to compare both approaches^[7]^. The aim of this study was to compare hospital and echocardiographic outcomes, survival, and reoperations after repeat transcatheter *versus* surgical mitral valve replacement.

## METHODS

### Design and Patients

**Fig. 1:**
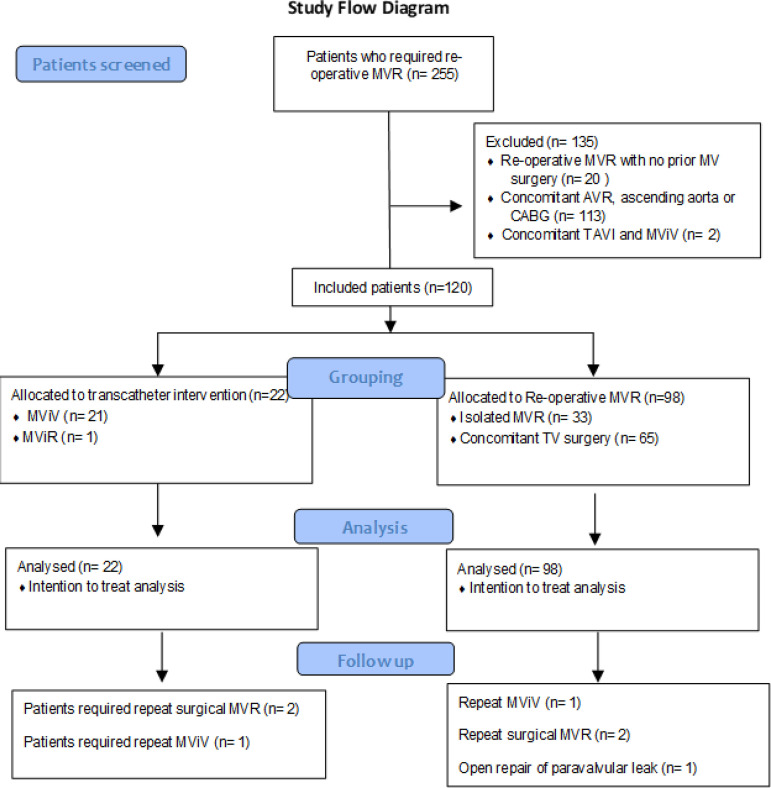
Study flowchart. AVR=aortic valve replacement; CABG=coronary artery bypass grafting; MVR=mitral valve replacement; MViR=mitral valve-in-ring; MViV=mitral valve-in-valve; TAVI=transcatheter aortic valve implantation; TV=tricuspid valve

We performed a retrospective study to compare patients who underwent rTMVR and rSMVR at Prince Sultan Cardiac Center, Riyadh, Saudi Arabia. The study included patients who underwent transcatheter mitral valve-in-valve (n=21) or mitral valve-in-ring (n=1) from March 2017 to July 2019 (Group 1). These patients were compared to patients who underwent rSMVR (n=98) with or without tricuspid valve surgery from April 2009 to October 2019 (Group 2). We excluded patients who underwent a concomitant transcatheter aortic valve replacement or other concomitant surgery and reoperative MVR without prior mitral valve surgery. The study flowchart is shown in Figure 1.

The Institutional Review Board of the Prince Sultan Cardiac Center approved the data collection for this study (Reference Number: R19022), and patients' consent to participate in the study was waived.

### Data Collection and Study Outcomes

Data were collected via paper and electronic medical records review. Preoperative data included patients' demographics, comorbidities, risk stratification using EuroSCORE II, preoperative renal function, left ventricular ejection fraction (LVEF), left ventricular end-diastolic diameter (LVEDD) and left ventricular end-systolic diameter (LVESD), and pulmonary artery systolic pressure (PASP).

All patients underwent pre- and postoperative echocardiography. Echocardiographic measurements were collected preoperatively, pre-discharge, and after 6, 12 and 18 months.

Study outcomes included in-hospital complications, intensive care unit (ICU) and hospital stay, cardiac readmissions, mitral valve reoperations, survival, and changes in echocardiographic measurements.

### Patient Assignment and Techniques

During the transcatheter mitral valve-in-valve era, patients were considered for this technique after heart team discussion. Patients who were eligible for surgery but refused surgical interventions were ofered the transcatheter option (n=8). Patients with infective endocarditis, mitral valve vegetations, left atrial thrombus, and those with a mitral valve size <25 mm were not considered for rTMVR. All patients underwent rTMVR via a transseptal approach, and our transcatheter mitral valve-in-valve technique was previously described^[8]^. Surgical mitral valve replacement was performed via median sternotomy in all patients.

Postoperative anticoagulation was similar in both groups. It included warfarin and acetylsalicylic acid (AAS) for three months, followed by life-long AAS unless patients had other indications for warfarin.

### Statistical Analysis: Data Presentation

Stata 16.1 (Stata Corp, College Station, Texas, USA) was used for all statistical analyses. We performed an intention-to-treat analysis to simulate clinical trials. Continuous data were presented as the 25^th^, 50^th^ (median), and 75^th^ percentiles. Normality was tested using the Shapiro-Wilk test, and the Wilcoxon rank-sum test was used to compare continuous variables. Chi-square test was used for categorical variables and, if the expected frequency was <5, Fisher’s exact test was used. We used the McNemar’s test to compare dependent categorical variables.

### Regression Models

Negative binomial regression was used to test the effect of the group and EuroSCORE II on postoperative hospital and ICU stay. Logistic regression analysis was used to identify the factors affecting hospital mortality, and Hosmer-Lemeshow and area under the curve were used to test the quality of the model.

Mixed-effects linear regression analysis was used to compare changes in the echocardiographic measurements between the two groups (LVEF, PASP, and mean mitral valve pressure gradient). The measurements were recorded at fixed times, preoperatively, pre-discharge, after 1 year, and after 18 months. The model yielded two values, the baseline measurements and the degree of change. The significance of the change was evaluated over time and compared between the two groups. The mixed-effect model included group, time, and baseline value.

### Time-to-Event Analysis

We compared three time-to-event variables (survival, reoperation, cardiac readmission) between the two groups. Kaplan-Meier method was used to plot the survival distribution for time-to-event variables, and the log-rank test was used to compare curves. Multivariable Cox regression was used to evaluate the effect of the surgical approach on time-to-event variables, and the proportional hazard assumption was tested using Schoenfeld residuals method.

## RESULTS

### Preoperative Data

Patients in Group 2 were significantly younger (72.5 [67-78] *vs.* 57 [52-64] years, *P*<0.001). Three (3.06%) patients had an implantable cardioverter-defibrillator in Group 2, and 1 (1.02%) patient underwent a previous transcatheter aortic valve implantation (TAVI). Mechanical valves were previously implanted in Group 2 in 23 (18.4%) patients. One (1.02%) patient in Group 2 had a hostile chest due to previous mastectomy and radiotherapy, 2 (2.04%) patients had peripheral artery disease, and 1 (1.02%) patient had a prior myocardial infarction. Seventeen (77.27%) patients in Group 1 and 64 (65.31%) patients in Group 2 have moderate or high tricuspid regurgitation (*P*=0.28). Preoperative data are presented in Table 1.

**Table 1:** Comparison of the preoperative characteristics and echocardiographic data between the two groups.

	Group 1 (n=22)	Group 2 (n=98)	*P*-value
Age (years)	72.5 (67-78)	57 (52-64)	<0.001
Females	15 (68.18%)	63 (64.29%)	0.729
Body mass index (kg/m^2^)	31.18 (25.22-34.89)	29.02 (26.12-33.39)	0.704
Permanent pacemaker	1 (4.55%)	11 (11.2%)	0.693
Previous PCI	1 (4.55%)	1 (1.02%)	0.334
Previous CABG	6 (27.27%)	19 (19.39%)	0.411
Number of previous surgeries			0.519
2	2 (9.09%)	10 (10.20%)	
3	2 (9.09%)	3 (3.06%)	
4	0	1 (1.02%)	
Previous stroke	1 (4.55%)	5 (5.10%)	>0.99
TIA	0	4 (4.08%)	>0.99
Diabetes mellitus	11 (50%)	32 (32.65%)	0.125
Hypertension	15 (68.18%)	43 (43.88%)	0.039
Smokers	0	6 (6.12%)	0.591
COPD	4 (18.18%)	7 (7.14%)	0.116
Chronic kidney disease	2 (9.09%)	8 (8.16%)	>0.99
ESRD on dialysis	0	3 (3.06%)	>0.99
HF within 2 weeks	6 (28.57%)	11 (11.22%)	0.078
NYHA III/IV	17 (77.27%)	64 (66.67%)	0.333
Cardiogenic shock within 24 hours	0	8 (8.16%)	0.348
Cardiomyopathy	2 (9.09%)	4 (4.08%)	0.303
Preoperative AF	12 (54.55%)	46 (47.92%)	0.575
EuroSCORE II	6.56 (5.47-8.04)	6.74 (4.28-11.84)	0.855
Creatinine (µmol/L)	76 (60-95)	76 (63-103)	0.646
LVEF (%)	55 (50-55)	55 (45-55)	0.118
LVEDD (mm)	45 (41-51)	49 (45-55)	0.026
LVESD (mm)	30.5 (27-36)	33 (29-39)	0.247
PASP (mmHg)	60 (55-80)	55 (45-70)	0.029
LV mass (g/m^2^)	160.7 (141.5-207.1)	178.4 (142.45-213.05)	0.502
Mitral regurgitation (moderate or greater)	16 (76.19%)	62 (63.27%)	0.644
Mitral stenosis	10 (47.62%)	38 (38.78%)	0.563
Mean pressure gradient (mmHg)	8.3 (6-11)	8.25 (5.3-11.9)	0.521

Continuous data are presented as median (25^th^-75^th^) percentiles and categorical data are presented as numbers and percentages. AF=atrial fibrillation; CABG=coronary artery bypass grafting; COPD=chronic obstructive pulmonary disease; ESRD=end-stage renal disease; HF=heart failure; LV=left ventricle; LVEDD=left ventricular end-diastolic diameter; LVEF=left ventricular ejection fraction; LVESD=left ventricular end-systolic diameter; NYHA=New York Heart Association; PASP=pulmonary artery systolic pressure; PCI=percutaneous coronary intervention; TIA=transient ischemic attack

### Operative and Postoperative Outcomes

Cardiopulmonary bypass time was 125 (104-159) minutes, and ischemia time was 90 (73-114) minutes. Implanted valve size was 26 mm (26-29) in Group 1 and 25 mm (25-27) in Group 2 (*P*=0.106). In Group 1, 2 (9.1%) patients underwent a concomitant tricuspid valve-in-valve implantation, in Group 2, 41 (41.84%) patients underwent a concomitant tricuspid valve (TV) repair, and 24 (24.49%) patients underwent a concomitant TV repair.

Postoperative complications are presented in Table 2. Patients in Group 1 had significantly shorter ICU and hospital stay. Pulmonary artery systolic pressure was lower in Group 2, and there was no diference in echocardiographic measures between the two groups (Table 3).

**Table 2:** Postoperative outcomes.

	Group 1 (n= 22)	Group 2 (n= 98)	*P*-value
New AF	3 (13.64%)	5 (5.1%)	0.161
PPM	2 (9.09%)	8 (8.16%)	>0.99
Endocarditis	0	3 (3.09%)	>0.99
LV OTO	1 (4.5%)	0	0.183
New renal impairment	2 (9.09%)	7 (7.14%)	0.669
Cerebral complications			0.337
TIA	0	1 (1.03%)	
Hemorrhagic stroke	1 (4.5%)	0	
Bleeding complications			0.002
Access-site bleeding	4 (18.18%)	7 (7.14%)	
GI bleeding	2 (9.09%)	0	
Major vascular complications	3 (13.64%)	3 (3.09%)	0.076
ICU stay (days)	1 (1-5)	3.5 (2-6)	0.013
Hospital stay (days)	4.5 (2-14)	14 (8-28)	<0.001
Hospital mortality	2 (9.09%)	7 (7.14%)	0.669

Continuous data are presented as median (25^th^-75^th^) percentiles and categorical data are presented as numbers and percentages. AF=atrial fibrillation; GI=gastrointestinal; ICU=intensive care unit; LVOTO=left ventricular outflow tract obstruction; TIA=transient ischemic attack

**Table 3:** Comparison of pre-discharge echocardiographic data.

	Group 1 (n=22)	Group 2 (n=98)	*P*-value
Discharge LVEF (%)	55 (50-55)	55 (45-55)	0.324
Discharge PASP (mmHg)	50 (45-60)	45 (35-50)	<0.001
Grade II mitral valve regurgitation	1 (5%)	0	0.183
Grade II paravalvular leak	0	1 (1.02%)	>0.99
Mean pressure gradient (mmHg)	6.5 (5.7-8.2)	6.1 (5-7.85)	0.240

LVEF=left ventricular ejection fraction; PASP=pulmonary artery systolic pressure

At discharge, 1 (5.56%), 4 (22.2%), 11 (61.1%), and 2 (11.1%) patients had tricuspid regurgitation grades 0, I, II and IV, respectively. In Group 2, 23 (30.7%), 30 (40%), 18 (24%), 1 (1.33%) and 3 (4%) patients had tricuspid regurgitation grades 0, I, II, III and IV at discharge, respectively (*P*=0.007).

### Predictors of Hospital Outcomes

ICU and hospital stays were significantly longer in Group 2 and with a higher EuroSCORE II. The groups did not affect the operative mortality. Mortality was higher with a higher EuroSCORE II (Table 4).

**Table 4:** Predictors of hospital and ICU stay (negative binomial regression with reporting coefficient) and hospital mortality (logistic regression with reporting odds ratio) (Hosmer-Lemeshow P=0.626; area under the ROC curve=0.706).

ICU stay	Coef./OR	*P*-value	95% Ci
Group 2	0.609	0.039	0.030-1.188
EuroSCORE II	0.054	0.002	0.019-0.088
**Hospital stay**			
Group 2	0.898	<0.001	0.434-1.363
EuroSCORE II	0.050	<0.001	0.026-0.074
**Hospital mortality**			
Group 2	(OR) 0.522	0.463	0.092-2.956
EuroSCORE II	(OR) 1.067	0.036	1.004-1.134

### One-Year Follow-Up

NYHA class improved significantly in both groups after one year compared to the preoperative value (*P*<0.001 for both groups). There was no diference in NYHA class between the two groups at 1-year follow-up (*P*=0.583).

### Changes in Echocardiographic Measurements

**Supplementary Fig. 1:**
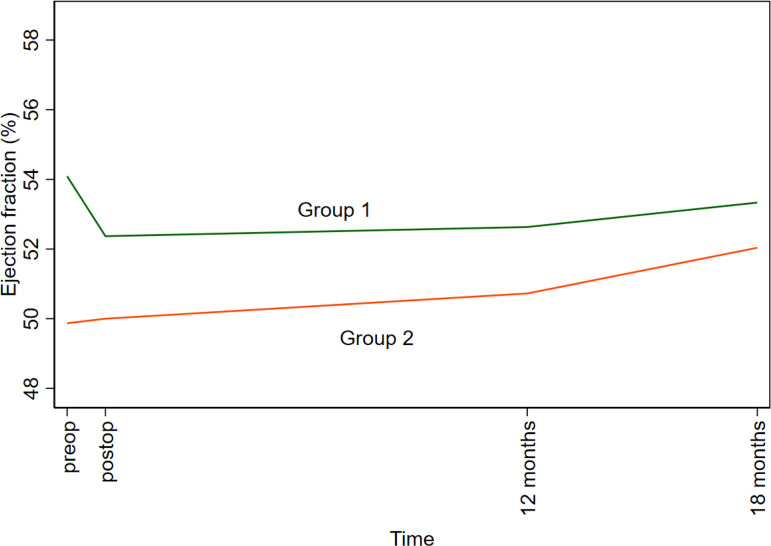
Changes in ejection fraction in both groups.

**Supplementary Fig. 2:**
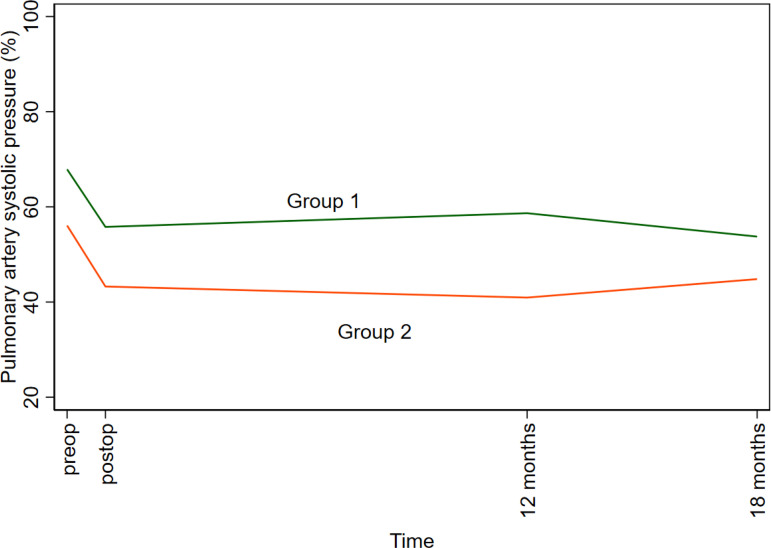
Changes in pulmonary artery pressure in both groups.

**Supplementary Fig. 3:**
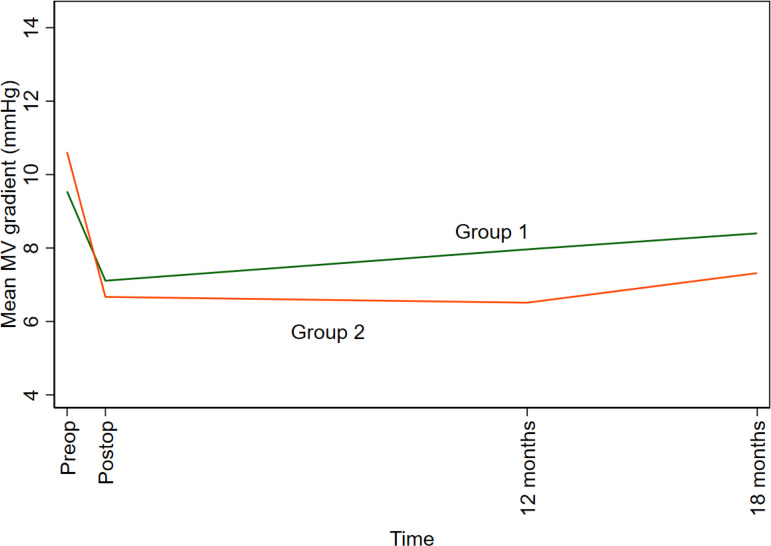
Changes in mean mitral valve gradient in both groups.

The groups did not infuence changes in LVEF, PASP, and mean mitral valve pressure gradient (Table 5) (Supplementary Figures 1-3).

**Table 5:** Mixed-effects REML regression for the changes in left ventricular ejection fraction, pulmonary artery systolic pressure, and mean mitral valve pressure gradient.

Ejection fraction	Coef.	*P*-value	95% CI
Group	−0.311	0.793	−2.637-2.015
Time	0.025	0.683	−0.097-0.148
Preoperative EF	0.601	<0.001	0.503-0.699
**PASP**			
Group	−3.039	0.153	−7.204-1.125
Time	−0.534	<0.001	−0.818 to −0.250
Preoperative PASP	0.596	<0.001	0.506-0.686
**Mean MV pressure gradient**			
Group	−0.226	0.841	−2.433-1.981
Time	−0.072	0.334	−0.219-0.074
Preoperative mean MV pressure gradient	0.501	<0.001	0.440-0.562

EF=ejection fraction; MV=mitral valve; PASP=pulmonary artery systolic pressure

### Time-to-Event Outcomes

**Fig. 2A-C:**
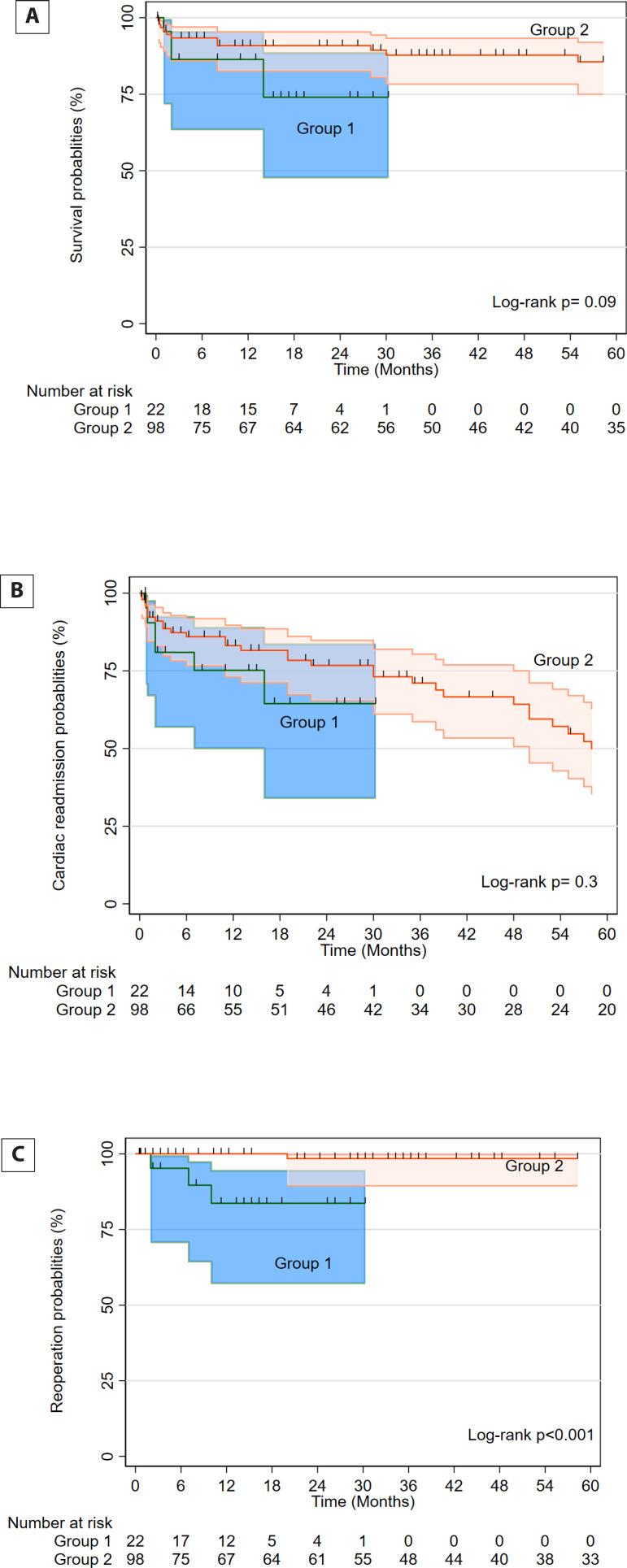
Kaplan-Meier distribution of survival, reoperation, and cardiac readmissions.

The median follow-up time was 28 (8-69) months; it was 15 (11-18) months in Group 1 and 36 (8-81) months in Group 2. Kaplan-Meier distribution of survival, reoperation and readmission for cardiac reasons are shown in Figures 2A, B, and C. Multivariable analysis showed no effect of the groups on survival or cardiac readmission (Table 6). However, reoperations were more frequent in Group 1. Three patients in Group 1 underwent reoperations: MVR and left atrial exclusion (n=1), MVR and left ventricular aneurysm repair (n= 1), repeat transcatheter mitral valve replacement (n=1). Four patients in Group 2 had reoperations: rTMVR and TAVI (n=1), repeat MVR for stuck valve (n=1), open repair of paravalvular leak (n=1), repeat MVR for degenerative valve (n=1).

**Table 6:** Multivariable Cox regression for factors affecting survival, cardiac readmission, and reoperation (proportional hazard assumption test P=0.948, 0.144 and 0.929).

Survival	HR	*P*-value	95% CI
Group	1.138	0.844	0.316-4.1
Age	1.083	0.002	1.029-1.141
**Readmission**			
Group	0.582	0.259	0.228-1.49
EuroSCORE II	1.030	0.126	0.992-1.070
**Reoperation**			
Group	0.042	0.01	0.004-0.474
Age	0.981	0.564	0.921-1.045

## DISCUSSION

Transcatheter mitral valve-in-valve replacement is an emerging new technology, which is considered as an alternative option to surgical reoperative MVR in patients with prohibitive or high surgical risk. The technique was listed in the European Society of Cardiology (ESC)/European Association for Cardio-Thoracic Surgery (EACTS) Guidelines (2017) as an alternative option for the management of degenerated bioprostheses in high-risk surgical patients^[9]^. We performed this study to compare rTMVR and rSMVR. Patients who underwent rTMVR were older and had higher PASP. Other preoperative variables, including EuroSCORE II, were comparable. There was no diference in operative mortality between the two groups, and the length of ICU and hospital stay was significantly shorter in rTMVR. We did not observe any significant diference in PASP, LVEF, and mean mitral valve pressure gradient changes over the follow-up between groups. Survival and cardiac readmission were similar in both groups; however, reoperation was significantly higher in patients who underwent rTMVR.

All patients in our rTMVR group had a transseptal approach, which played an important role in decreasing the ICU and hospital stay^[10]^. Additionally, this approach was associated with a lower bleeding rate than the transapical approach^[11,12]^. Computed tomography (CT) scan was not required for planning the transcatheter approach but was an essential part of the preoperative evaluation before rSMVR. No dye was used during rTMVR, and the ring of the mitral valve prosthesis was used to localize the valve. EuroSCORE II was comparable between groups, which can be explained by including 8 patients in the rTMVR group with low EuroSCORE who refused to undergo surgery.

We did report a significant diference in operative mortality, similar to the findings of Kamioka et al.^[7]^. They reported a 30-day mortality of 3.2% after rTMVR and 3.2% after rSMVR, which is lower than our results. Our mortality is within the range reported in the literature^[13,14]^. In the Society of Thoracic Surgeons' annual report, the in-hospital mortality in high-risk patients who underwent transcatheter mitral valve-in-valve was 7.2%. The 30-day mortality was 8.5%^[15]^, which is comparable to that of those who underwent transcatheter mitral in our results. In a meta-analysis of transcatheter mitral valve-in-valve procedures, the 6-month mortality was 23%^[16]^, and it was 13.5% in our study. The non-significant diference in hospital mortality in our series could be attributed to the comparable EuroSCORE II between groups, which was a significant predictor of mortality. Two-year survival was 74% and 90% in rTMVR and rSMVR groups, respectively. However, this diference did not reach statistical significance.

The mean mitral valve pressure gradient was not diferent between groups both at discharge and during follow-up. This includes patients who underwent a mechanical or bioprosthetic mitral valve replacement. The mean mitral valve pressure gradient reported in our series was comparable to several reports^[5,7,11]^. Since the transcatheter procedure was valve-in-valve, a higher pressure gradient was expected. However, patient selection may contribute to the non-significant diference between the two groups. The transcatheter approach was not used in patients with small valves (<27 mm), making patient-prosthesis mismatch a low probability.

No studies to our knowledge have compared the long-term outcomes after rTMVR and rSMVR. In the present study, we found that both approaches improved clinical symptoms with no diference in survival and cardiac readmission between groups. However, patients who underwent rTMVR had a higher rate of reoperation. The high incidence of reoperation in this group could be attributed to the learning curve since most of these operations were required early. Five patients who underwent rSMVR required reoperation at a median follow-up of 36 months compared to 15 months in patients who underwent rTMVR. Conclusion about the potential earlier degeneration of transcatheter valves cannot be drawn from our study, and further studies are required.

Our study showed that the outcomes of rSMVR and rTMVR are comparable. Both techniques improved clinical outcomes and patients' symptoms. Patients who had left atrial thrombus and endocarditis, in addition to those with small implanted valves, should be considered for surgical MVR. A randomized trial is recommended to compare both approaches in patients who are considered to be at high risk for surgery.

### Limitations of the Study

The main limitation of our research is the retrospective nature of the study. Patients assigned to each group were diferent, and the assignment was confounded by indication. However, we performed a multivariable regression analysis for the main variables that may affect the outcomes. Another limitation is the shorter follow-up period, which is attributed to the recent introduction of the transcatheter approach. The sample size is relatively small, but we created a restricted cohort study by applying rigid inclusion criteria for surgical and transcatheter approaches. Patients who had concomitant procedures, apart from tricuspid valve reintervention, were excluded. This was essential to decrease heterogeneity between the studied groups. Lastly, the two groups had an unequal number of patients, which could have affected the significance of several variables.

## CONCLUSION

Transcatheter mitral valve-in-valve can shorten ICU and hospital stay compared to repeat surgical mitral valve replacement with a comparable mortality rate. rTMVR is a safe procedure; however, it has a higher risk of reoperation. rTMVR can be an option in selected high-risk patients. Furthermore, larger clinical randomized studies are required to confirm these findings

## References

[1] Gaur P, Kaneko T, McGurk S, Rawn JD, Maloney A, Cohn LH (2014). Mitral valve repair versus replacement in the elderly: short-term
and long-term outcomes. J Thorac Cardiovasc Surg.

[2] David TE, Armstrong S, McCrindle BW, Manlhiot C (2013). Late outcomes of mitral valve repair for mitral regurgitation due
to degenerative disease. Circulation.

[3] Anyanwu AC, Itagaki S, Varghese R, Castillo J, Chikwe J, Adams DH (2014). Re-repair of the mitral valve as a primary strategy for early and
late failures of mitral valve repair. Eur J Cardiothorac Surg.

[4] Keenan NM, Newland RF, Baker RA, Rice GD, Bennetts JS (2019). Outcomes of redo valve surgery in indigenous
Australians. Heart Lung Circ.

[5] Yoon SH, Whisenant BK, Bleizifer S, Delgado V, Schofer N, Eschenbach L (2017). Transcatheter mitral valve replacement for degenerated
bioprosthetic valves and failed annuloplasty rings. J Am Coll Cardiol.

[6] Ejiofor JI, Hirji SA, Ramirez-Del Val F, Norman AV, McGurk S, Aranki SF (2018). Outcomes of repeat mitral valve replacement in patients with
prior mitral surgery: a benchmark for transcatheter
approaches. J Thorac Cardiovasc Surg.

[7] Kamioka N, Babaliaros V, Morse MA, Frisoli T, Lerakis S, Iturbe JM (2018). Comparison of clinical and echocardiographic outcomes after
surgical redo mitral valve replacement and transcatheter mitral
valve-in-valve therapy. JACC Cardiovasc Interv.

[8] Otaiby MA, Al Garni TA, Alkhushail A, Almoghairi A, Samargandy S, Albabtain M (2020). The trans-septal approach in transcatheter mitral valve-in-valve
implantation for degenerative bioprosthesis. J Saudi Heart Assoc.

[9] Baumgartner H, Falk V, Bax JJ, De Bonis M, Hamm C, Holm PJ (2017). 2017 ESC/EACTS guidelines for the management of valvular heart
disease. Eur Heart J.

[10] Bouleti C, Fassa AA, Himbert D, Brochet E, Ducrocq G, Nejjari M (2015). Transfemoral implantation of transcatheter heart valves after
deterioration of mitral bioprosthesis or previous ring
annuloplasty. JACC Cardiovasc Interv.

[11] Eleid MF, Whisenant BK, Cabalka AK, Williams MR, Nejjari M, Attias D (2017). Early outcomes of percutaneous transvenous transseptal
transcatheter valve implantation in failed bioprosthetic mitral valves, ring
annuloplasty, and severe mitral annular calcification. JACC Cardiovasc Interv.

[12] Seifert M, Conradi L, Baldus S, Schirmer J, Knap M, Blankenberg S (2012). Transcatheter mitral valve-in-valve implantation in patients with
degenerated bioprostheses. JACC Cardiovasc Interv.

[13] Vohra HA, Whistance RN, Roubelakis A, Burton A, Barlow CW, Tsang GM (2012). Outcome after redo-mitral valve replacement in adult patients: a
10-year single-centre experience. Interact Cardiovasc Thorac Surg.

[14] Jaussaud N, Gariboldi V, Grisoli D, Berbis J, Kerbaul F, Riberi A (2012). Risk of reoperation for mitral bioprosthesis
dysfunction. J Heart Valve Dis.

[15] Grover FL, Vemulapalli S, Carroll JD, Edwards FH, Mack MJ, Thourani VH (2017). 2016 annual report of the society of thoracic surgeons/American
college of cardiology transcatheter valve therapy registry. J Am Coll Cardiol.

[16] Hu J, Chen Y, Cheng S, Zhang S, Wu K, Wang W (2018). Transcatheter mitral valve implantation for degenerated mitral
bioprostheses or failed surgical annuloplasty rings: a systematic review and
meta-analysis. J Card Surg.

